# Mutual mother‐pup acoustic identification in Asian particolored bats

**DOI:** 10.1002/ece3.9554

**Published:** 2022-11-22

**Authors:** Xiao Tan, Yu Li, Keping Sun, Longru Jin, Jiang Feng

**Affiliations:** ^1^ Jilin Provincial Key Laboratory of Animal Resource Conservation and Utilization Northeast Normal University Changchun China; ^2^ Jilin Provincial Engineering Laboratory of Avian Ecology and Conservation Genetics Northeast Normal University Changchun China; ^3^ College of Life Science Jilin Agricultural University Changchun China

**Keywords:** acoustic individual signature, acoustic recognition, echolocation pulse, isolation calls, playback experiment, *Vespertilio sinensis*

## Abstract

In many vertebrates, vocal communication is crucial in parent–offspring interactions, and parents are often able to discriminate between the calls of their own and others' offspring. There are many reports on the unidirectional recognition of isolation calls of pups by maternal bats, but few studies on the ability of bat pups to recognize maternal acoustic signals. In this study, we investigated whether the echolocation pulses of female Asian particolored bats (*Vespertilio sinensis*) and isolation calls of pups differ statistically among individuals. We used two‐choice playback experiments to test whether the mothers and pups of *V. sinensis* can recognize each other by acoustic signals. Both the echolocation pulses of mother bats and the isolation calls of pups contained sufficient individual characteristics. Playback experiments showed that mothers were able to recognize isolation calls of pups, and most pups greater than 12 days old were able to distinguish echolocation pulses of their own mother from those of other mothers. This is the first use of two‐choice acoustic signal playback experiments to confirm that pups can recognize their mothers by echolocation calls. The results provide behavioral evidence for bidirectional recognition of acoustic signals between mothers and infants in frequency‐modulated type bats.

## Graphical abstract

This is the first use of two‐choice acoustic signal playback experiments to confirm that pups can recognize their mothers by echolocation calls. The results provide behavioral evidence for bidirectional recognition of acoustic signals between mothers and infants in frequency‐modulated type bats. The study may deepen our understanding of bat acoustic signal function and adaptive evolution under selection pressure.

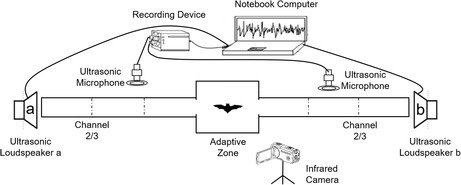

## 1 INTRODUCTION

Evolutionary theory predicts that birds and mammals breeding in colonies can discriminate between their own and other offspring and direct parental care to their own progeny (Blank & Yang, 2017). Fitness theory suggests that parental reproduction investment should be concentrated on offspring to improve their fitness. Effective recognition reduces unnecessary parental energy consumption and ensures adequate energy supply during subsequent parental reproduction (Jin et al., 2015). The offspring recognizing their parents, in turn, facilitates parent–offspring reunions in group breeding species and reduces the probability of offspring being attacked by non‐kin parents. For example, some adult Australian sea lions exhibit aggressive reactions to food solicitation by unrelated young (Gwilliam et al., 2008). In mammals, parent–offspring recognition is mostly unidirectional (i.e., parents recognize their own offspring), but a few mammals, e.g., narwhal, *Monodon Monoceros* (Ames et al., 2021); harbor seal, *Phoca vitulina* (Ravignani et al., 2019); and sheep, *Ovis aries* (Morton et al., 2018), have bidirectional recognition between parents and their infants.

Bats (Chiroptera) are the only mammals capable of flight. Most insectivorous bats live in highly clustered groups and rely relatively little on vision due to their nocturnal lifestyles (Chaverri et al., 2018). Bats rely on acoustic signals for orientation and communication (Bradbury & Vehrencamp, 1976; Brown, 1976b; Gould, 1971; Jones et al., 2021). Compared to vision, bat acoustic signals are less influenced by obstacles and have no light limitations (Forrest, 1994). These signals are an important means to mediate parent–infant recognition (Jin et al., 2015). In bats, the majority of parental care is provided by females (Knörnschild et al., 2013) and mother bats selectively nurse only their own offspring (Jin et al., 2015; Knörnschild & von Helversen, 2008). To facilitate maternal identification, bat pups are able to emit isolation calls containing individual identity information shortly after birth (Luo et al., 2020; Wilkinson, 2003). Mother bats, in turn, produce acoustic signals to response the vocalization of their pups or to locate pups during mother–pup interactions (Balcombe, 1990; DeFanis & Jones, 1996; Jin et al., 2015; Knörnschild & von Helversen, 2008).

Most female bats can recognize their own offspring using the isolation calls emitted by pups (Balcombe & Mccracken, 1992; Knörnschild & von Helversen, 2008). For example, the big brown bat, *Eptesicus fuscus* (Gould, 1971), the pallid bat, *Antrozous pallidus* (Brown et al., 1978), the Seba's short‐tailed bat, *Carollia perspicillata* (Knörnschild et al., 2013) and the Geoffroy's bat, *Myotis emarginatus* (Mehdizadeh et al., 2021), can recognize their offspring through isolation calls with distinct vocal signatures. Some studies have suggested the existence of bidirectional acoustic recognition between mothers and infants, e.g., the little brown bat, *Myotis lucifugus* (Gould, 1971); the greater horseshoe bat, *Rhinolophus ferrumequinum* (Matsumura, 1979); and the Pale spear‐nosed bat, *Phyllostomus discolor* (Esser & Schmidt, 1989), but acoustic playback experiments have not been conducted to test this possibility. Most playback experiments have found that pups could not recognize the acoustic signals of their mothers (Balcombe & Mccracken, 1992; DeFanis & Jones, 1996; Knörnschild & von Helversen, 2008). However, only a few playback experiments have confirmed the bidirectional recognition of acoustic signals between mothers and infants in bats. For example, in a study of mother–infant interactions in the vespertilionid bat, *Plecotus auratus* (DeFanis & Jones, 1995a), the pups were able to recognize the calls of their mothers. Jin et al. (2015) found that pomona leaf‐nosed bats (*Hipposideros pomona*) had bidirectional acoustic recognition between mothers and offspring. Considering that mutual mother‐pup acoustic identification has only been shown in a few bat species, studies are needed in more bat species.

The Asian particolored bat (*Vespertilio sinensis*) is distributed in Russia, Mongolia, Korea, Japan, and China (Fukui et al., 2019). They usually occur in clusters in bridge cavities or in the roofs of old buildings (Fukui et al., 2010). During the nonmating season, males and females live separately, with adult females forming breeding groups with their pups (Jin et al., 2012) and adult males forming separate male clusters or living alone (Luo et al., 2017). Each summer, about 1500 female Asian particolored bats and their pups roost in clusters under a highway bridge in Acheng, Heilongjiang Province, northeastern China (Zhao et al., 2019). As bat pups grow and develop, their mobility increases and there is usually no distance between individuals (field observations). In this situation, when female bats return from foraging, it may be difficult for female bats and infants to accurately locate each other using spatial memory and/or smell (Swift, 1981). However, acoustic recognition may have significant advantages because the acoustic signals of female bats and the isolation calls of pups usually contain sufficient individual characteristics for recognition (Balcombe & Mccracken, 1992; Esser & Schmidt, 1989; Gelfand & McCracken, 1986; Kunz & Hood, 2000). Therefore, acoustic recognition may play an important role in bat mother–pup recognition. The aim of this study was to determine if bidirectional recognition between mothers and infants of *V. sinensis* can be accomplished using acoustic signals. We hypothesized that both mothers and bat pups can recognize each other by acoustic signals and that the recognition is bidirectional. First, we predicted that the calls of both mothers and offspring have individual characteristics with statistically significant differences. Second, the mother bats were predicted to be able to recognize the pups by their isolation calls. Finally, we predicted that the infants could recognize calls emitted by their mother.

## 2 MATERIALS AND METHODS

### 2.1 Collection and rearing of experimental individuals

On June 25, 2018, 20 female *V. sinensis* in late pregnancy (determined by abdominal touch; Kunz & Fenton, 2005) were captured under the Acheng District overpass in Harbin, Heilongjiang Province. The individuals were marked with marking rings (4.2 mm internal width, 5.5 mm height, Porzana Ltd.) on their forearms and then placed in a temporary laboratory (4 × 3 × 3 m) about 2.3 km away from the sampling site. In the laboratory, the ambient temperature (26 ± 2°C), relative humidity (55 ± 5%), and light (lights on: 05:00–16:00; lights off: 16:00–05:00) were set to simulate the environmental parameters of the natural habitat of *V. sinensis*. Bats were able to fly freely in the temporary laboratory. In the early stage, female bats were trained to actively look for food (yellow mealworms) and water until all females could feed independently. We regularly ground vitamin and calcium tablets into powder and added them to the yellow mealworms' feeding box so that the mealworms could feed on these supplements. We observed females hourly for the birth of pups. Two infrared cameras (MI, CMSXJ03C, China, with external monitors in other rooms) were used for observation purposes. Once a bat birth was observed, we put marker rings on the pups and recorded their sex. Mother–infant pairs were placed in different cages with same size (60 × 70 × 80 cm), and the pups were fed and cared for by mother bats. A total of 30 pups (15 females and 15 males) were produced by 20 female bats (10 had twins, and the other 10 had one pup).

### 2.2 Sound recording and analysis

**FIGURE 1:**
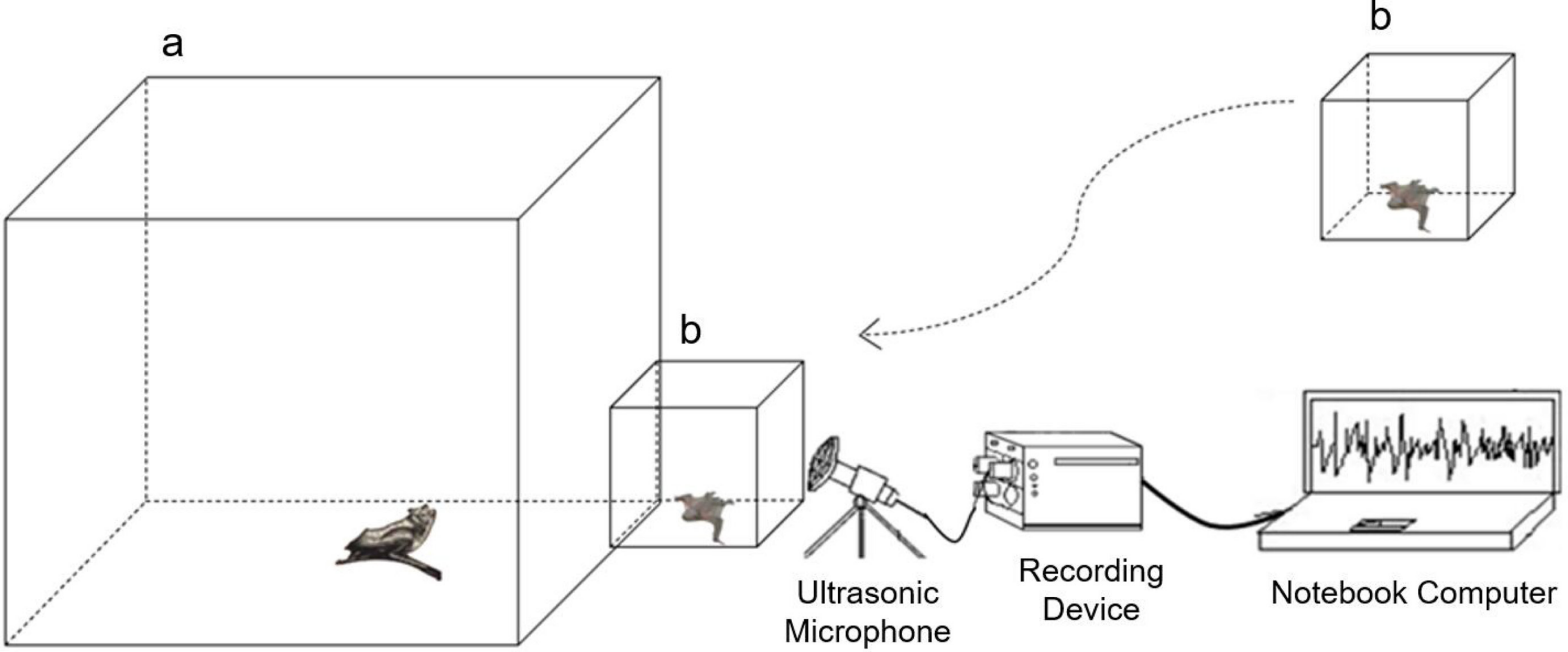
Maternal call recording trials.

To record echolocation pulses and/or directive calls of mother bats during mother–infant reunions, the mother bat was placed in a customized cage a (60 × 60 × 60 cm; Figure 1). When it was acclimated to the environment (i.e., no searching crawling and vocal behavior), a smaller experimental cage b (25 × 25 × 25 cm) with the pup was placed beside experimental cage a. An ultrasonic microphone (Ultrosoundgate CM16/CMPA, Avisoft Bioacoustics) was placed directly facing the entrance of cage b to record maternal calls during the mother–pup reunion. The microphone was connected to an ultrasound recording interface with a sample rate of 250 kHz at 16‐bit resolution, and every 60 s generated a wave file that was stored in a notebook computer. During this process, we only recorded echolocation pulses from mother bats. Therefore, subsequent experiments were carried out on the echolocation pulses of female bats.

To ensure the health of the pups, isolation calls were not recorded during the first two days of life and were started 2 days later. When pups were separated from mothers, they were placed and recorded in a 3 × 2.5 × 3 m chamber surrounded by sound‐attenuating foam. The average recording time of each young bat was about 2 min, and the maximum time did not exceed 5 min. An ultrasonic microphone (UltroSoundGate CM16/CMPA, Avisoft Bioacoustics) was 1 m away from the mouth and nose of the focal pup. The sampling frequency was 250 kHz, the resolution was 16 bits, and every 60 s generated a wave file. The pups were encouraged to vocalize by gently stroking their backs. We were certain that isolation calls, not distress calls, were elicited by pups because their spectrograms were different. Disposable gloves were worn during the operation to avoid the influence of odor from the experimenter's hand skin.

Twenty high signal‐to‐noise ratio echolocation pulses (>30 dB) were randomly selected from each female bat (400 calls for the 20 females). We analyzed maternal echolocation pulses with Avisoft‐SASLab Pro version 5.1 (Avisoft Bioacoustics). Prior to conducting acoustic analysis, the call was standardized to 75% to evaluate the quality of the waveforms and exclude overloaded signals (Mumm et al., 2014; Sun et al., 2018). Measurements were taken from spectrograms generated using a 1024‐point fast Fourier transformation and a Hamming window with 75% overlap.

To characterize variation in echolocation pulses uttered by mothers, two temporal parameters—duration and interval—and four spectrum‐based parameters—peak frequency, minimum frequency, maximum frequency and bandwidth—were measured from the harmonic containing the most energy of the call. These parameters are important and commonly used for determining whether the acoustic signals have an individual signature (Jin et al., 2015; Knörnschild et al., 2013; Knörnschild & von Helversen, 2008). For these four spectrum‐based parameters, four locations (i.e., start, end, center, and maximum amplitude of the element) were measured (Balcombe & Mccracken, 1992; Gelfand & McCracken, 1986; Gillam & Chaverri, 2012). Thus, a total of 19 acoustic variables were extracted, namely duration, interval, root‐mean‐square (rms), peak frequency (start), minimum frequency (start), maximum frequency (start), bandwidth (start), peak frequency (end), minimum frequency (end), maximum frequency (end), bandwidth (end), peak frequency (centre), minimum frequency (centre), maximum frequency (centre), bandwidth (centre), peak frequency (maximum), maximum frequency (maximum), minimum frequency (maximum), and bandwidth (maximum). Similarly, the isolation calls of 5‐day‐old pups were analyzed. Isolation calls are usually multiharmonic in structure, and the main energy is concentrated in the first harmonic. Therefore, only the first harmonic (fundamental frequency) was selected for acoustic parameter measurement. Twenty calls were randomly selected from each pup (600 calls for 30 pups), and 19 acoustic parameters mentioned above were selected for analysis.

### 2.3 Sound editing and playback

The recorded echolocation pulses of mothers were edited using Avisoft‐SASLab Pro (Version5.1). For each female bat, we randomly selected five series of pulse trains, each 50 ms, that had a high signal‐to‐noise ratio, and then we combined these randomly and assembled them in accordance with natural intervals in Avisoft‐SASLab Pro 5.1 to obtain playback sequences that were 70 s long, i.e., 30 s of echolocation pulses + 10 s of silent interval + 30 s of echolocation pulses. The recorded isolation calls of pups were edited in the same way to generate a new playback file. The editing method was 30 s isolation calls + 10 s silent interval + 30 s isolation calls.

**FIGURE 2:**
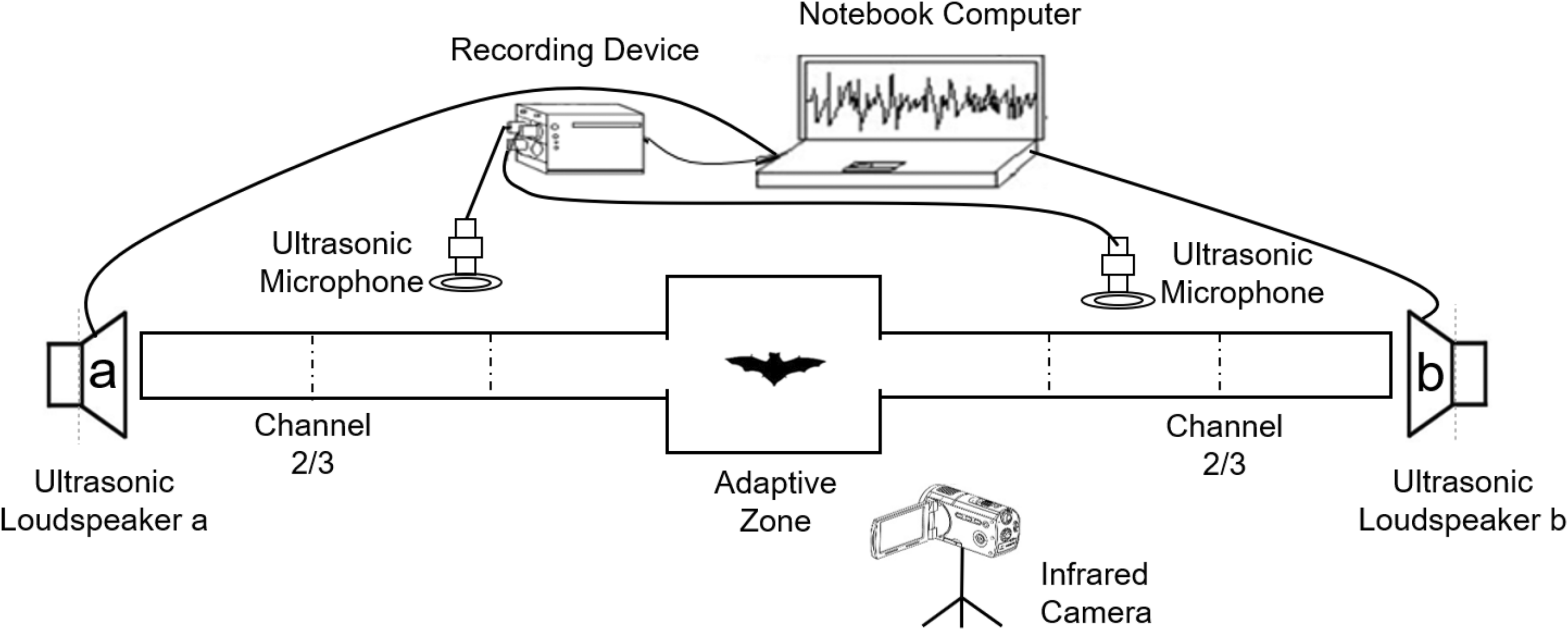
Experimental set‐up for two‐choice acoustic playback.

In the acoustic experiment of female bats recognizing their pups, the female bats were placed in the central adaptation area of the test arena, and two channels allowing bats to crawl on were extended on both sides of the central adaptation area (Figure 2 shows the experimental setup). After they had acclimatized to the environment (no exploration behavior, no crawling and no echolocation pulses in two minutes), an Ultrasonic loudspeaker (ScanSpeak, Avisoft Bioacoustics) was used to playback the edited acoustic files of kin and non‐kin pups on both sides of the experimental channel at the same time. The placement of sounds from kin and non‐kin pups was randomized on both sides, and each time a new mother bat was tested. An infrared camera (HDR‐CX760E, Sony Corp) and ultrasound recording interface (Avisoft UltraSoundGate116H, Avisoft Bioacoustics) were used to record behavioral responses (i.e., whether the mother crawls in the direction of the calls of the kin pup) and echolocation pulses of experimental female bats, respectively (Figure 2). The recording device had a sampling frequency of 250 kHz at 16‐bit resolution. During the experiment, excluding the adaptation time of the experimental individual in the adaptation area, the behavioral response was recorded, and the recording time was 70 s (duration of playback file). The video recording was stopped after the playback file finished playing. It was stipulated that the experimental individual would prefer left or right side's calls after climbing out of the adaptation area and beyond 2/3 positions of the channel (Figure 2). We speculated that this is possibly due to recognition. For each bat, echolocation pulse was played back twice, but to exclude individual influences, only the data from the first experiment were used in the analysis.

In the experiment of pups recognizing maternal calls, pups aged 12–21 days (two at 12 days of age, four at 14 days of age, 12 at 16 days of age, eight at 19 days of age, and four at 21 days of age) were selected for playback experiments. Pups younger than 12 days of age were usually small and weak and showed little crawling behavior (personal observation). It was therefore difficult to determine whether they could recognize maternal echolocation pulses by the behavioral response of crawling. The experimental procedures were similar to that of females identifying pups. Each pup was tested only once, and 30 pups (15 females and 15 males) were subjected to 30 playback experiments. The playback of both its own mother and alien mothers' acoustic stimuli simultaneously on both sides of the apparatus avoided the phonotaxis behavior and excluded the behavioral responses of experimental individuals that were simply due to curiosity about the acoustic signals (Balcombe & Mccracken, 1992).

### 2.4 Statistical analysis

To reduce the multicollinearity between the acoustic variables of the original maternal echolocation pulses, all the measured parameters were analyzed by principal component analysis (PCA) in SPSS 22.0 (IBM Corp.). Four principal components (eigenvalues > 1) were extracted from the dimensionality reduction of 19 parameters, which explained 81.1% of the total variance. The first principal component (PC1) contributed 46.6% and large values were associated with bandwidth (maximum; 0.98), bandwidth (centre; 0.98), bandwidth (start; 0.95) and bandwidth (end; 0.92), suggesting that they greatly contributed to PC1. The second principal component (PC2) contributed 20.6% of the total variation. Large values were associated with peak frequency (maximum; 0.87) and peak frequency (centre; 0.86). The third principal component (PC3) and the fourth principal component (PC4) contributed 8.1% and 5.8%, respectively. For PC3 and PC4, all acoustic parameters had low contribution (all absolute values < 0.8). For pup isolation calls, four principal components (eigenvalues > 1) were extracted, which together explained 71.1% of the total variance. PC1 contributed 36.5% and large values were associated with maximum frequency (maximum; 0.86), maximum frequency (centre; 0.84) and bandwidth (maximum; 0.80). PC2 contributed 20.4% and large values were associated with minimum frequency (centre; 0.85) and minimum frequency (maximum; 0.84). PC3 and PC4 contributed 8.3% and 6.0%, respectively, and all acoustic parameters had low contribution (all values < 0.8). The PCA results from maternal echolocation pulses and pup isolation calls indicated that frequency and bandwidth may play important roles in acoustic characteristics. The four principal components extracted from echolocation pulses of mother bats and isolation calls of pups were conducted using discriminant function analyses (DFA) to determine if individuals may be discriminated based on acoustic parameters. The significance of classification success was compared with chance classification using a binomial test. We observed the video, recorded the frequency of bats choosing kin and non‐kin mother echolocation pulse directions, and compared the differences with random classification probabilities using exact binomial tests (Jiang et al., 2016). The analysis of results showed whether bidirectional recognition between mothers and infants can be achieved by acoustic signals.

### 2.5 Ethical note

All rearing as well as experimental procedures were in accordance with the ASAB/ABS Guidelines for Treatment of Animals in Behavioral Research and were approved by the Wildlife Conservation Office of the Jilin Forestry Department, China (approval number: NENU‐W‐2014‐101). Bat capture methods and experiments in this study conformed to the Northeast Normal University guidelines for animal behavior research. The female bats were allowed to fly freely in the temporary laboratory for 2 h each night before being returned to the mother–infant pair cage. After 12 days of age, the pups showed obvious flapping behavior. The mother and infant pairs, except for the experimental individuals on that day, were allowed to practice flying in the temporary laboratory for 3 h per day to ensure that the mother bats and pups have normal flying ability after the experiments. Each bat was examined individually before the experiment every day. All the bats were put into the simulated ecological environment laboratory the night before release and allowed to fly and live freely. After one night of adaptation, the health of the bats was judged according to their vigorous behavior and their fur condition before being released back to the habitat the next day. No deaths occurred during the feeding and experimental period (26 days).

## 3 RESULTS

**FIGURE 3:**
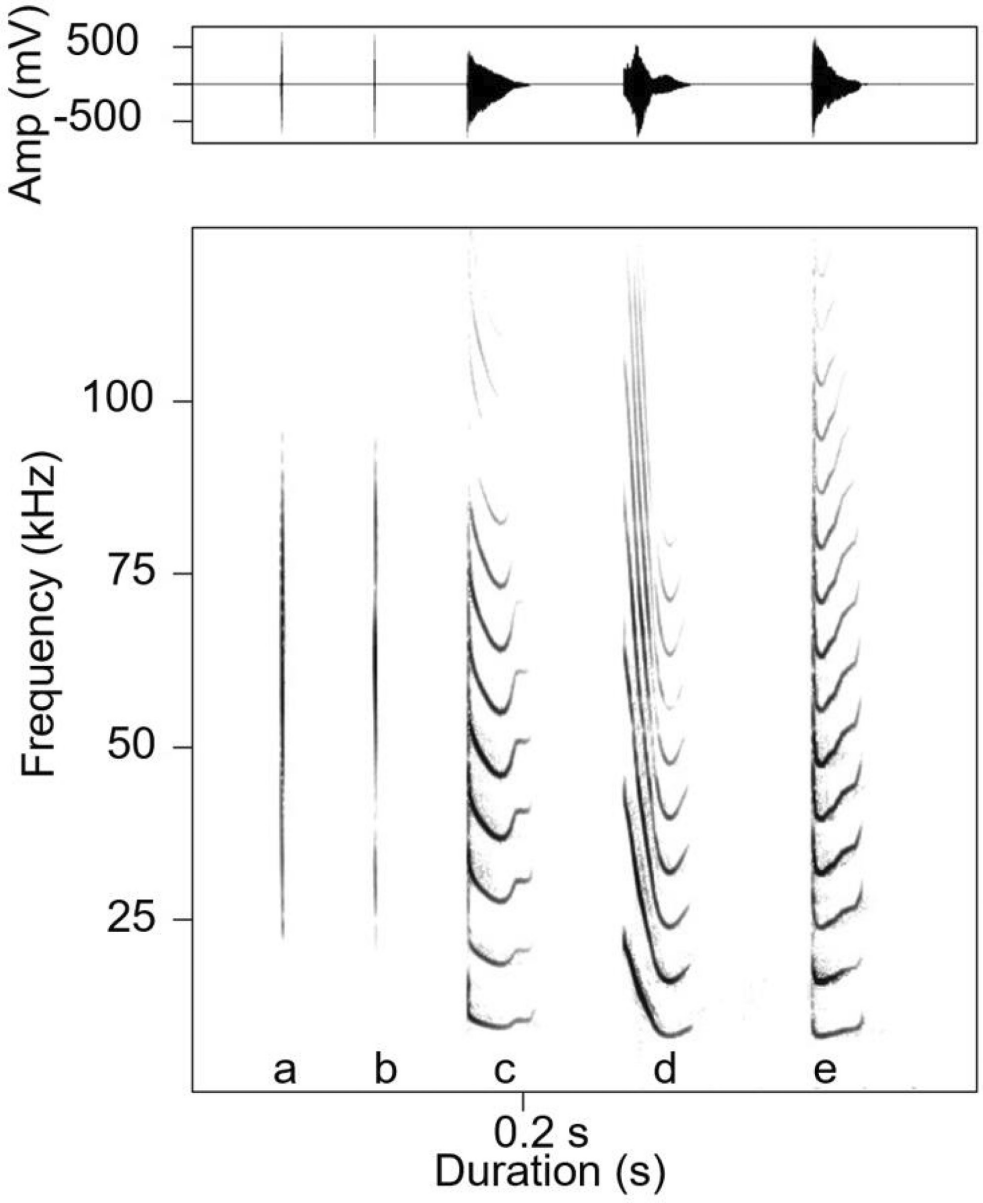
Oscillograms (above) and spectrograms (below) of a mother's echolocation pulses (a, b) and isolation calls of three pups (c–e) of *Vespertilio sinensis*. All spectrograms were produced using a 1024‐point FFT and a hamming window with 75% overlap.

Discriminant function analysis (DFA) was performed on 400 echolocation pulses of 20 female bats (see Figure 3, calls a, b). The DFA results showed that 23.3% of the calls were classified to the correct individual, which was significantly higher than expected by chance (5.0%; binomial test: *p* < .001). Discriminant function 1 and function 2 together explained 90.9% of the observed variation (Table 1). The DFA with 600 isolation calls syllables (see Figure 3, calls c, d, e) from 30 pups classified 35.9% to the correct individual, which was significantly higher than expected by chance (3.3%; binomial test: *p* < .001). Discriminant function 1 and function 2 together explained 88.9% of the observed variation (Table 2).

**TABLE 1:** Statistical evidence for an individual signatures in echolocation pulses of female *Vespertilio sinensis*.

Discriminant function	Eigenvalue	% of variance	Wilks's lambda	*p*
1	0.871	60.6	0.329	<.001
2	0.436	30.3	0.615	<.001
3	0.107	7.4	0.883	.054
4	0.023	1.6	0.977	.919

**TABLE 2:** Statistical evidence for an individual signature in isolation calls of *Vespertilio sinensis* pups.

Discriminant function	Eigenvalue	% of variance	Wilks's lambda	*p*
1	4.580	65.0	0.035	<.001
2	1.687	23.9	0.195	<.001
3	0.549	7.8	0.523	<.001
4	0.236	3.3	0.809	<.001

In acoustic recognition experiments of pups by mother bats, female bats were played back 20 times, 19 of which the females climbed toward their own pups and stayed at the loudspeaker at the end of the experimental channel with emitting echolocation pulses. In all experiments, only one female bat only crawled in the middle adaptation area of the experimental set‐up and did not show a clear preference for the playback calls played on either side. Females selected the calls of their own pups more frequently than they did to calls of non‐kin pups (Exact binomial tests: *p* < .01, *N* = 20).

In acoustic recognition experiments of mother bats by pups, 26 of 30 pups climbed toward their mothers side and four pups climbed in the direction of the non‐kin female bat. One of these four pups was a female and three are males. The frequency of infants choosing the calls of kin female bats was significantly higher than that of non‐kin female bats (Exact binomial tests: *p* < .01).

## 4 DISCUSSION

### 4.1 Individual signature of acoustic signals

Consistent with previous studies (DeFanis & Jones, 1996; Gould, 1975; Luo et al., 2020; Wilkinson, 2003), our results showed that both the echolocation pulses emitted by mother bats and isolation calls emitted by pups contain sufficient individual differences for individual recognition. Some studies in FM bats showed that one or two of the parameters of bandwidth, amplitude, initial frequency, and duration contribute the most in individual identification of echolocation pulses (Esser & Schmidt, 1989; Voigt‐Heucke et al., 2010; Yovel et al., 2009). In our study, the contribution in the discrimination from maternal echolocation calls and pup isolation calls both come from bandwidth and frequency, which is consistent with previous studies (Gelfand & McCracken, 1986; Mehdizadeh et al., 2021; Scherrer & Wilkinson, 1993).

In addition, compared to some studies (Balcombe, 1990; DeFanis & Jones, 1995a; Knörnschild & von Helversen, 2008), the correct classification of echolocation pulses and isolation calls was low in our study. However, as suggested by Müller and Manser (2008) and Mumm et al. (2014), a low DFA classification result on individual calls does not imply the lack of individual recognition. Most mammals encode their acoustic individual characteristics with source characteristics such as frequency, amplitude contours and harmonic structure (Mumm et al., 2014). In this study, we only measured some parameters from the harmonic containing the most energy of the calls, which could not really represent the entire acoustic differences among individuals. Perhaps the use of other measures, such as cepstral coefficients and amplitude contours, might help to understand these differences better.

Although bat echolocation pulses are used for orientation and navigation, many studies have found that echolocation pulses have individual signatures (Brown, 1976a; Esser & Schmidt, 1989; Jones & Siemers, 2011; Yovel et al., 2009) and can also encode information (ranging from group affiliation to individual identity; Ancillotto & Russo, 2014; Dorado‐Correa et al., 2013; Kazial et al., 2008). After hearing the calls from the mother bat, the pups will emit isolation calls (Engler et al., 2017; Kunz & Fenton, 2005; Wilkinson, 2003). Isolation calls have sufficient individual characteristics to reflect individual differences (Gelfand & McCracken, 1986; Mehdizadeh et al., 2021; Scherrer & Wilkinson, 1993) and female bats can identify their own pups though the differences (Wilkinson, 2003).

### 4.2 Maternal bats recognition of isolation calls made by their pups

In this study, almost all females climbed toward their infants, indicating that females were able to recognize the isolation calls made by their pups. This is consistent with the results in previous studies showing that females can clearly distinguish their own offspring and alien offspring based solely on isolation calls. Females respond more frequently and with shorter reaction times to their own offspring (Balcombe & Mccracken, 1992; Bohn & Gillam, 2018; DeFanis & Jones, 1996). In this study, only one female did not climb in the direction of its own pups. Actually, in the process of raising mother–infant pairs of individuals, we found that the female bat abandoned her young pups many times, and the young bats were often separated from the mother and could not be breastfed.

### 4.3 Recognition of echolocation pulses by pups to their mother bats

The majority of pups in this study climbed in the direction of the maternal bat's calls, indicating that pups can recognize the mother bats through the individual characteristics of echolocation pulses. Four pups failed to recognize mother calls, two at 12 days of age and two at 14 days of age. However, all infants older than 14 days successfully recognized the echolocation pulses of their mother. Based on related studies on the auditory development of infant bats from other species (Brown et al., 1978; Matsumura, 1979), we believe that the auditory system of *V. sinensis* pups older than 14 days in this study may be complete so that they had the ability to recognize individual characteristics of echolocation pulses of adult bats.

### 4.4 Bidirectional recognition of acoustic signals between mothers and infants

In most vertebrates, the recognition between mother and infant is unidirectional (Blank & Yang, 2017; Prat et al., 2017). For cluster‐feeding birds and mammals, many factors such as large population size, enhanced mobility of offspring, and prolonged mother–infant separation have increased the selection pressure (Briefer & McElligott, 2011; Charrier, 2020) and promoted bidirectional recognition between mothers and offspring. For example, razorbills (*Alca torda*) are a highly gregarious species of seabird that provides biparental care to their chicks in the nest after which the male is the sole caregiver for a period at sea. Once the chicks become active and leave the nest to join the male at sea, identifying the young is challenging and promotes two‐way identification of parents and offspring (Insley et al., 2003).

In studies of maternal–infant recognition in bats, some acoustic recognition results have shown that maternal–infant recognition in bats is unidirectional, with only the mothers actively recognizing their young (Balcombe & Mccracken, 1992; DeFanis & Jones, 1996; Esser, 1998; Knörnschild & von Helversen, 2008). A possible reason for this result is that the selection pressure on the bat species studied was not great. Although they are distributed in groups, individuals have their own territorial space, and other clues can be used in the process of mother–infant recognition, such as olfaction (Liang et al., 2021), touch, and spatial memory (Gustin & McCracken, 1987). Other studies have explored whether offspring can recognize their mother's acoustic signals (Balcombe & Mccracken, 1992; DeFanis & Jones, 1995b; Esser, 1998). For example, the constant‐frequency type of pomona leaf‐nosed bats (*H. pomona*) can elicit bidirectional recognition between mothers and infants (Jin et al., 2015). *Vespertilio sinensis* use bidirectional recognition of acoustic signals between mothers and infants because of the strong selection pressure on mothers and infants. It is difficult for mothers to use spatial memory to find their pups because the offspring may climb to other places. Olfaction is also likely to be insufficient for a successful mother–pup reunion because it is usually used by bats for closed‐up recognition (Liang et al., 2021; Mccracken, 1993).

Echolocation pulses are mostly used for navigation and detection (Neuweiler, 1990; Schnitzler et al., 2003), but they also have a communication function (Schuchmann & Siemers, 2010). However, the subjects of these studies were adult bats, and less has been reported about bat infants whether they can recognize their mothers' acoustic signals. Regarding the role of maternal echolocation pulses in maternal–infant recognition, echolocation pulses of the greater sac‐winged bat have been played back to offspring, but the pups indiscriminately vocalized in response to echolocation pulses from their own and alien mothers (Knörnschild & von Helversen, 2008). Although reciprocal recognition has been shown in the FM bat *Myotis lucifugus* (Turner et al., 1972), it was not possible to determine whether recognition was based on auditory and/or olfactory stimuli. In our study, acoustic playback experiments provided behavioral evidence for bidirectional recognition of acoustic signals between mothers and infants in *V. sinensi*.

In conclusion, we used statistical analysis and playback experiments of maternal and infant acoustic signals in *V. sinensis* to demonstrate that *V. sinensis* uses bidirectional recognition of maternal and infant acoustic signals. Mothers can recognize their pups by pup isolation calls, and pups can recognize the specific echolocation pulses of their mothers. This study increases our understanding of bat acoustic signals functions and their adaptive evolution under selection pressure.

## AUTHOR CONTRIBUTIONS

**Xiao Tan:** Conceptualization (equal); data curation (equal); formal analysis (lead); investigation (equal); methodology (lead); visualization (lead); writing – original draft (lead). **Yu Li:** Investigation (equal). **Keping Sun:** Supervision (equal). **LongRu Jin:** Conceptualization (equal); funding acquisition (equal); project administration (lead); supervision (lead); writing – review and editing (lead). **Jiang Feng:** Funding acquisition (supporting); resources (lead).

## CONFLICT OF INTEREST

The authors declare that they have no competing interests.

## Data Availability

If our paper is accepted for publication, data used in this study will be archived on the Dryad Digital Repository.
